# Auditory and cognitive performance in elderly musicians and nonmusicians

**DOI:** 10.1371/journal.pone.0187881

**Published:** 2017-11-29

**Authors:** Massimo Grassi, Chiara Meneghetti, Enrico Toffalini, Erika Borella

**Affiliations:** Department of General Psychology, University of Padova, Via Venezia 8, Padova, Italy; Baycrest Health Sciences, CANADA

## Abstract

Musicians represent a model for examining brain and behavioral plasticity in terms of cognitive and auditory profile, but few studies have investigated whether elderly musicians have better auditory and cognitive abilities than nonmusicians. The aim of the present study was to examine whether being a professional musician attenuates the normal age-related changes in hearing and cognition. Elderly musicians still active in their profession were compared with nonmusicians on auditory performance (absolute threshold, frequency intensity, duration and spectral shape discrimination, gap and sinusoidal amplitude-modulation detection), and on simple (short-term memory) and more complex and higher-order (working memory [WM] and visuospatial abilities) cognitive tasks. The sample consisted of adults at least 65 years of age. The results showed that older musicians had similar absolute thresholds but better supra-threshold discrimination abilities than nonmusicians in four of the six auditory tasks administered. They also had a better WM performance, and stronger visuospatial abilities than nonmusicians. No differences were found between the two groups’ short-term memory. Frequency discrimination and gap detection for the auditory measures, and WM complex span tasks and one of the visuospatial tasks for the cognitive ones proved to be very good classifiers of the musicians. These findings suggest that life-long music training may be associated with enhanced auditory and cognitive performance, including complex cognitive skills, in advanced age. However, whether this music training represents a protective factor or not needs further investigation.

## Introduction

Professional musicians have attracted much attention in recent years (e.g. [1–3]) because they show sensory, motor, and cognitive abilities, which are often better than in their nonmusician peers. Some authors suggest that the music training drives functional and structural brain plasticity (see [4, 5]), and for this reason musicians are often regarded as a model for studying plasticity across the lifespan (e.g. [1–3]).

### Musicians and nonmusicians: Auditory and cognitive performance

Musicians outperform nonmusicians in many auditory tasks. The former are more proficient in recognizing melodies that have been transposed [6], or reproduced in a faster or slower tempo [7], they are better at detecting mistuned notes [8, 9], and they are also better in several classic psychoacoustical tasks involving supra-threshold sound processing. For example, musicians outperform nonmusicians in frequency [10], temporal [11] or spectral discrimination [12]. Notably, the musicians’ superiority extends to speech as well: they are better at recognizing the prosody of a sentence [13, 14], and understanding speech in noise [15]. Differences between musicians and nonmusicians also emerge with bio-recording techniques: musicians have more pronounced event-related potentials associated with the perception of irregularities in musical syntax [9, 16], and with the processing of pitch and pitch patterns (e.g. [9, 17–21]).

Musicians perform better than nonmusicians in several cognitive domains and in several cognitive tasks too, such as in memory tasks (e.g. [22–24]). One of the crucial mechanisms of cognition is working memory (WM), or the ability to simultaneously maintain and process information, and this has also been found enhanced in musicians (e.g. [23]). Professional musicians exhibited shorter reaction times than nonmusicians in a non-musical auditory attention task, indicating a greater ability to focus their attention (e.g. [25]). They proved more efficient than nonmusicians in other higher-level cognitive processes too, including executive functions (e.g., inhibiting cognitive control) or processing speed [25–27]. Musicians showed also enhancement of visual abilities in comparison to nonmusicians, specifically in visuospatial tasks (e.g. [28–31]). For instance, Pietsch and Jansen [32] found that young music students (practicing music more than eight hours a week) had stronger visuospatial (rotation) skills than education science students.

Not all studies have shown that musicians perform better in cognitive tasks, however. Some found similar WM performance between musicians and nonmusicians (e.g. [33–34]); and it has been reported that musicians performed no better than nonmusicians in short-term memory tasks, reasoning, or executive functions [35]. Several authors reported finding of not enhanced spatial abilities in musicians either (e.g., Children: [36]; Adults: [37, 38]). The above-mentioned results thus show that auditory performance is enhanced in musicians by comparison with nonmusicians, while the picture is less clear as regards the former’s potentially better cognitive performance.

The above studies nonetheless prompt an important question concerning music and aging. What happens in older musicians? Do the perceptual and cognitive advantages of musicians over nonmusicians persist in aging? Since playing a musical instrument requires the multimodal integration of sensory, motor, and cognitive information, continuous practice and repetition of such skills over a long period of time might contribute to cognitive functioning and, as recently suggested, to the reorganization of functioning in several brain areas (e.g. [39]).

### Older adult musicians and nonmusicians: Auditory and cognitive performance

Whether older adults are musicians or not, aging coincides with an elevation of the absolute hearing threshold, particularly at higher frequencies. This phenomenon is called presbycusis, i.e., age-related hearing loss (e.g. [40–42]). Supra-threshold sound processing also deteriorates with age (e.g. [43, 44]). The elderly’s performance in auditory tasks declines, i.e., they have higher difference limens in frequency, intensity or duration discrimination [43, 45–50]. Older adults’ performance also deteriorates in the temporal processing of sounds, i.e., in their sinusoidal amplitude-modulation detection thresholds (SAM), gap detection thresholds [42, 51–55], and spectral discrimination (e.g. [43]—but see also [56] who found no evidence of this). A musician’s supra-threshold sound processing ability deteriorates with age too, but life-span studies suggest that musicians retain a better auditory performance than nonmusicians in several psychoacoustic tasks because they can draw on their greater abilities in the frequency or temporal dimensions and in understanding speech in noise [40]; see [57] for an overview. Older musicians fare better than old nonmusicians in detecting a mistuned harmonic when listening to a complex tone stimulus [58], for instance, and they are faster at speech sound classification [59].

However, only few studies have considered the cognitive profile of older musicians and compared it with that of nonmusicians. Age-related changes in cognitive abilities are well documented (e.g. [60–62]), but little is known about whether practicing music benefits cognition in old age, and which (if any) aspects of cognition it may sustain, and to what degree. Some studies found that older musicians scored higher than older nonmusicians in verbal memory tests (i.e., immediate recall) and executive processes [63, 64]. A greater efficiency in certain aspects of cognitive control, such as conflict resolution, and control over irrelevant information, has also been reported in elderly musicians compared with their nonmusician peers [65]. As for WM, in three of the four studies that involved complex WM tasks, the authors found that the elderly musicians performed better in this area too [27, 64, 65]; but see [63]. When visuospatial short-term memory tasks were used, on the other hand, the differences between elderly musicians and nonmusicians were not significant [63, 64].

It is therefore worth further investigating the cognitive profile of older musicians and see whether they have (or not) better cognitive performances than nonmusicians in aging. In addition, the previously-mentioned studies included individuals who could hardly be defined as ‘older adults’ as some were 45 years of age [27], or under 64 years old ([63–65]; 50–77 years-old). The studies also considered different levels of musical expertise: some musicians had 10 or more years of experience [63], while others had begun their musical training before age nine and had played a musical instrument throughout their lives [27].

### The present study

For the first time (to our knowledge, at least), the present study investigated both the auditory and the cognitive performance of professional musicians over 65 years of age, comparing them with their nonmusicians peers. In terms of hearing, both peripheral auditory processing and supra-threshold auditory processing were assessed. The functioning and performance of peripheral auditory processing is thought to coincide with a listener’s absolute threshold, and are usually assessed by means of pure tone audiometry [40–42]. Supra-threshold processing was investigated, on the other hand, by assessing participants’ basic auditory abilities (e.g. see [43, 66–67]), i.e., their ability to discriminate the frequency, intensity, and temporal and spectral dimensions of an acoustic stimulus, which coincide with the main dimensions of human sound perception [66]. We postulated that the performance of older musicians and nonmusicians would differ not in terms of the peripheral processing of sounds, i.e., in absolute threshold (e.g. [40–42]), but in auditory abilities (e.g. [40]).

To measure cognitive performance, different short-term memory indicators, complex WM tasks, and a measure of executive functioning (inhibition) were administered. Visuospatial abilities were investigated too, as according to some studies seem to be enhanced in musicians (e.g. [32, 31]). Like WM, visuospatial skills are age-sensitive, have a strong impact on older adults’ quality of life, and have proved to be a core factor when it comes to analyzing the cognitive profile across the adult life-span [68]. We investigated in particular whether any superiority of musicians over nonmusicians is especially evident in tasks that are age-sensitive, and that weigh on attentional resources, i.e., in the complex and higher-order tasks [61, 69, 70]. In fact, the advantage of musicians over nonmusicians in short-term and WM tasks has been found identical in young adults (see [71]). There is also evidence of musicians and nonmusicians differing very little in short-term memory tasks (e.g. [63, 64]), whereas the former appear to perform better than the latter in WM tasks [27, 64, 65]. If WM abilities are intensively involved in the training to become a musician, in older age we might expect larger group differences in WM tasks than in short-term memory tasks. This issue is especially important because it has been well documented in the aging literature that WM tasks requiring higher attentional control are more severely affected by the age-related decline [72]. Since some memory models (see [73]) indicate that task content affects performance in tasks demanding more attentional control/resources, i.e., WM tasks, verbal (presented auditorily) and visuospatial WM tasks were also administered to our participants in order to explore whether any superiority in the musicians’ WM task performance depended on their content or not. We also assessed the performance of musicians and nonmusicians in visuospatial tests and inhibition measures. These are complex tasks and there are reports in the literature of musicians showing a more developed visual imagery than nonmusicians [31, 32], and more efficient inhibitory mechanisms [65].

Finally, because sensory (e.g., auditory) performance and cognitive performance deteriorate with age, a corollary aim of the present study was to examine the relationship between the auditory and cognitive decline, in both musicians and nonmusicians (see [74] for a recent overview). Other studies comparing musicians with nonmusicians suggest that enhanced auditory performance might be associated with an enhanced level in certain cognitive skills, supporting the classic hypothesis of a relationship between sensory performance and cognitive performance (e.g. [43, 75–78]). A study comparing elderly lifelong musicians with age-matched nonmusicians on auditory and various cognitive tasks might shed some light on the possible link between these two domains.

## Method

### Participants

The study involved 40 older adults, comprising 20 professional musicians (six females, 65 ≤ age ≤ 84) and 20 nonmusicians (six females, 65 ≤ age ≤ 84). All participants were Italian native speakers and volunteered for the study. The musicians had between 46 and 80 years of music training and practice (*M* = 60.30, *SD* = 9.96), and they were still actively playing, solo or in an orchestra. Eight played string instruments (the violin in 7 cases, the cello in 1), eight played keyboard instruments (the piano in 5, the organ in 3), and four played other instruments (harp, flute, oboe, and trumpet). Participants were screened for cognitive impairments with the short version of the Italian Checklist for the Multidimensional Assessment (SVAMA) of the elderly used in the Veneto region [79]. None of them showed any signs of cognitive dysfunction, such as mild cognitive impairment or Alzheimer’s disease, and they all answered the 10 items on the SVAMA correctly, indicating a good cognitive functioning. None of them scored below the age- and education-matched norms in the WAIS-R [80] vocabulary test, which is a measure of crystallized intelligence (Italian norms [81]). Three one-way ANOVAs revealed that the musicians and nonmusicians did not differ in age (*F* < 1). The nonmusicians had significantly more years of formal education than the musicians, however, *F*(1, 39) = 60.03, *p* < .001, *n*_*p*_^*2*^ = .62, and a higher score in the WAIS-R vocabulary test, *F*(1, 39) = 16,76, *p* < .001, *n*_*p*_^*2*^ = .31 (see Table 1).

**Table 1:** Demographic variables by group (musicians vs nonmusicians).

	Musicians		Nonmusicians	
	*M*	*SD*	*M*	*SD*
Age	72.25	6.71	72.70	6.71
Years of education	9.40	3.02	15.85	2.18
Vocabulary	46.40	5.49	55.90	8.81

### Auditory tasks

The tasks were administered using a laptop computer connected to an M-AUDIO FastTrack Pro sound card. The output from the sound card was presented diotically through Sennheiser HD 280 headphones. Sounds were synthesized in real time at a sample rate of 44.1 kHz with a 24-bit resolution. The tests were always conducted in quiet rooms (i.e., noise level at the participant’s ear below 35 dBA). The sounds used to measure the participants’ auditory abilities were presented at 70 dB SPL.

The hearing experiments were written in a custom-coded MATLAB program (MathWorks, Natick, MA) using two free custom MATLAB toolboxes [82, 83]. Pure tone audiometry was done separately for the left and right ears, and for frequencies of 500, 1500, and 4000 Hz. The tone duration was 1 sec. The threshold was estimated with one block of trials for each frequency. In each block, the tone intensity was set at a comfortable level for trial 1, then manipulated according to a simple up-down rule [84]. In each trial, participants were asked to report whether they could hear the tone or not. Threshold tracking was continued up to the fourth reversal, and the final threshold was calculated by averaging the thresholds at the four reversal points. After establishing the absolute threshold, participants completed six tests within a single listening session. These tests were selected as being representative of the three main non-speech auditory abilities identified by the factorial analysis conducted by Kidd and colleagues [66]: “loudness and duration”, represented here by an intensity discrimination test and a duration discrimination test; “amplitude modulation”, represented here by a sinusoidal amplitude modulation (SAM) detection test, and by a gap detection test; and “pitch and time”, represented here by a frequency discrimination test and a spectral shape discrimination test. Participants completed two blocks of twenty trials for each test. In each trial, they were presented with three sound intervals separated by a 500 ms silence. Two intervals were identical (the standards), while one (the variable) differed in one acoustic characteristic (oddball paradigm). In the frequency discrimination test, for example, the frequency of the variable interval was a certain amount of delta higher than that of the standards. After each trial, participants were asked to say which of the three intervals was the variable one. The order of presentation of the standards and variable intervals was randomized before each trial. For the first trial in each block, delta was set so as to make the trial easy for participants. Delta was varied according to a simple up-down rule in trials 1 to 10 [84], then according to a maximum likelihood algorithm in trials 11 to 20 [85]. In the present study, the maximum likelihood algorithm tracked the 66%-correct point of a participant’s psychometric function. The threshold was calculated by averaging the thresholds returned by the maximum likelihood algorithm in trial 20 of the two blocks. Each auditory ability task is described in detail below.

#### Frequency, intensity and duration discrimination

The standard intervals were two 1 kHz pure tones lasting 500 ms, and gated on and off with two 10 ms raising cosine ramps. The variable interval was identical to the standard tone except for a higher frequency (or higher intensity, or longer duration). The frequency, intensity and duration of the variable were allowed to home in on a participant’s discrimination threshold within a range of 1000.1–1500 Hz, 500.1–900 ms, and 0.01–15 dB, respectively.

#### Spectral shape discrimination

The standard intervals were two, 500 ms long complex tones including the first five harmonics of a 333.3 Hz fundamental frequency, and they were gated on and off with two 10 ms raising cosine ramps. All harmonics were of identical amplitude, and they were added in phase so that the spectral centroid of the complex tone was 1 kHz. The variable interval was identical to the standards except that the third harmonic was on a higher level than the others, which made the timbre of the standards and variable different. The level of the third harmonic of the variable interval was allowed to home in on a participant’s discrimination threshold within a range of 0.1 to 30 dB higher than the level of the other harmonics.

#### Gap detection and SAM detection

The standard intervals were two identical Gaussian noises lasting 500 ms, gated on and off with two 10 ms raising cosine ramps. For gap detection, the variable interval was identical to the standards except that its temporal center was cleared to create the gap. The gap was gated on and off with two 0.5 ms raising cosine ramps. The duration of the gap was allowed to home in on a participant’s detection threshold within a range of 0.1–64 ms. For SAM detection, the variable interval was identical to the standards except that the amplitude was modulated by a 10 Hz sinusoidal modulator. The modulation depth was allowed to home in on a participant’s threshold within a range of -60 to 0 dB (no modulation).

### Cognitive tasks

#### Short-term memory tasks

*Forward and backward Corsi tasks* (adapted from Corsi [86]). These tests consist of a set of nine blocks randomly placed on a wooden tablet. The cubes are numbered on the experimenter's side of the board to facilitate the administration of the test. The experimenter taps the blocks in a random sequence, and participants are asked to reproduce the sequence in the same (forward Corsi) or in reverse order (backward Corsi). The difficulty of the task is manipulated by increasing the number of blocks tapped by the experimenter. The sequences are presented at a rate of one cube per second. The tests started with 3 and increased to 8 cubes in each sequence in the forward version, and from 2 to 7 in the backward version. Each level of difficulty included 2 sequences of the same length. After two consecutive recall errors, the task was discontinued. A practice trial with two sequences was administered for each type of task before the test started. One point was awarded for each correctly recalled sequence. The final score corresponded to the total number of correctly-recalled sequences (maximum score 12, for both tasks).

#### Complex (visuospatial and verbal) working memory tasks

*Visual Pattern Test Active* (VPTA; adapted from [87]; see [70]). In this task, participants were presented with a matrix in which half the cells were filled; these matrices increased in size from the smallest (4 squares with 2 cells filled) to the largest (20 squares with 10 cells filled). Then participants were given a blank matrix and asked to fill in the cells to reproduce the same pattern as on the matrix they had seen, but one row lower down. For example, if the second cell in the first row of the presentation matrix had been filled, participants had to fill in the second cell in the second row. For scoring purposes, each pattern was attributed a value according to its complexity. The final score was obtained from the sum of the values for the three most complex patterns correctly completed.

*Listening Span Test* (LST; see [60], adapted from [88]). This verbal task consists of an increasing number (2, 3, 4, 5, 6) of simple sentences. The task consisted of 20 sentences in all in which each sentence was separated from the next by an interval of 1.5 sec. Each sentence can contain from 6 to 12 words. The last word of each sentence can be composed of 2, 3, 4, or 5 syllables. Participants were asked to listen to each sentence, judge its plausibility (say whether it was true or false) and remember the last word. At the end of each set, participants were asked to recall all the last words, in their order of presentation. Two training trials preceded the task. The total number of last words correctly recalled in the right order during the whole test was considered as the measure of a participant’s WM capacity. The number of intrusion errors (words recalled that had been heard during the task but were not the last words of a sentence) was also computed. This procedure was used to measure a participant’s ability to control the permanence of information in their WM (e.g. [89]).

#### Visuospatial abilities

The *short Embedded Figures Test* (sEFT, adapted from [90]; see also [91]) consists of 10 items, each comprising a complex overall figure in which participants were asked to identify an embedded simple shape shown separately in a list of simple figures. The *short Mental Rotations Test* (sMRT, adapted from [92]; see also [91]) consists of 10 items, each involving a 3D target figure (assembled cubes) presented with four possible matches alongside. Participants were asked to find the two figures that were identical to the target but rotated in space (time limit for the task 5 min). Participants’ accuracy was calculated from the scores obtained in the two tasks (sEFT and sMRT; 1 point for each correct answer, max 10 points for each task.

#### Procedure

Participants were tested individually in two separate sessions lasting about 90 minutes each, with a one-week interval between the two sessions to avoid familiarity effects on tasks measuring the same construct. In the first session, participants completed a health and demographic questionnaire followed by the SVAMA, the sMRT, and the auditory tasks, presented in the following order: pure tone audiometry, SAM detection, duration discrimination, frequency discrimination, gap detection, intensity discrimination and spectral shape discrimination. In the second session, the task order was: VPTA, sEFT, Forward and Backward Corsi tasks, and LST. The LST was presented verbally, while all the other tasks (sMRT, sEFT, VPTA) were administered as “paper and pencil” tests. For the latter, the experimenter ascertained that participants could read the instructions and stimuli with ease, then provided examples, and ensured that the requirements of the task were understood, before proceeding with the actual task. The current research was approved by the Ethic committee of the University of Padova for psychological research (protocol n. 1966). All participants gave their informed written consent to participate in the study.

## Results

### Auditory tasks

**Fig 1:**
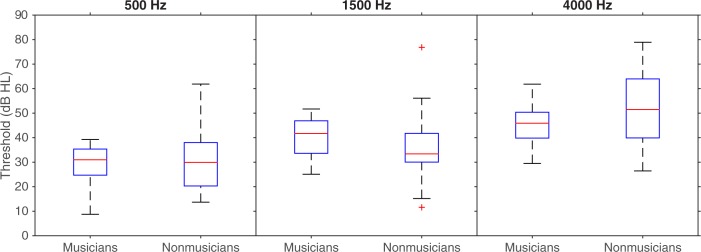
Boxplots representing the pure tone audiometry of participants separately for each ear and by group. In each box, the central mark is the median. The edges of the box are the 25^th^ and 75^th^ percentiles. The whiskers are the interquartile range (i.e., Q3-Q1) augmented by 50%, and the symbols are outliers.

**Fig 2:**
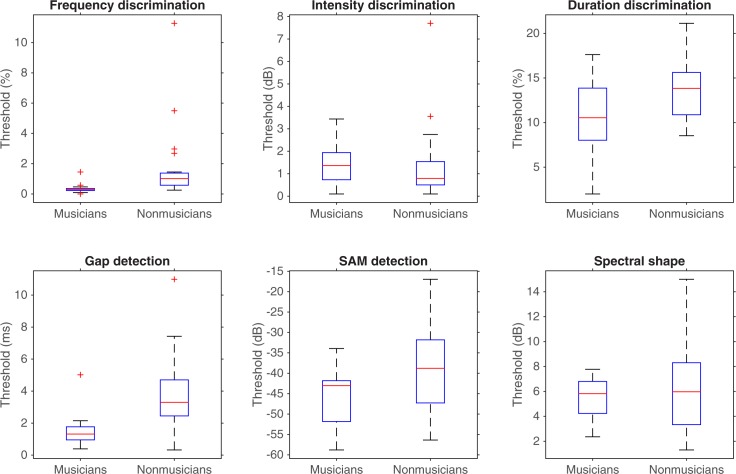
Boxplots representing the auditory performance of participants in the psychoacoustic tests. In each box, the central mark is the median. The edges of the box are the 25^th^ and 75^th^ percentiles. The whiskers are the interquartile range (i.e., Q3-Q1) augmented by 50%, and the symbols are outliers.

Musicians and nonmusicians were compared in terms of their performance on auditory measures with a series of analyses of variance (ANOVA). Individual participants’ pure-tone audiometry results were averaged across frequency and ears. Musicians and nonmusicians did not differ in terms of absolute thresholds, *F*(1,38) = .04, *p* = .84, *η*^*2*^_*p*_ < .01 (see Fig 1). Musicians performed better than nonmusicians in: the frequency discrimination task, *F*(1,38) = 6.61, *p* = .01, *η*^*2*^_*p*_ = .15; the duration discrimination task, *F*(1,38) = 5.05, *p* = .03, *η*^*2*^_*p*_ = .12; gap detection, *F*(1,38) = 19.57, *p* < .001, *η*^*2*^_*p*_ = .34; and SAM detection, *F*(1,38) = 5.89, *p* = .02, *η*^*2*^_*p*_ = .13. The two groups’ performance was similar in: the intensity discrimination task, *F*(1,38) < .01, *p* = .96, *η*^*2*^_*p*_ < .01; and spectral shape discrimination, *F*(1,38) = 1.18, *p* = .28, *η*^*2*^_*p*_ = .03. The results of the psychoacoustic tests are represented in Fig 2.

### Cognitive tasks

**Fig 3:**
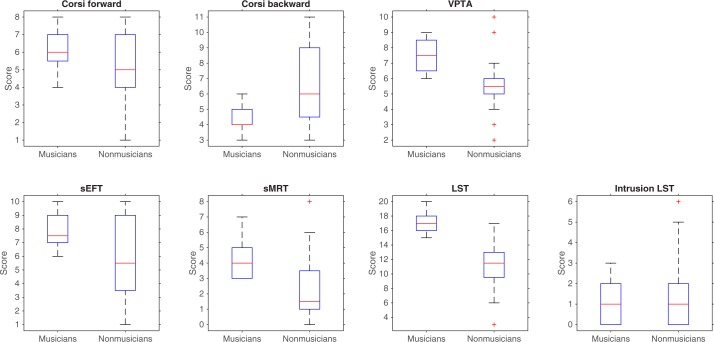
Boxplots representing the cognitive performance of participants in the cognitive tests. In each box, the central mark is the median. The edges of the box are the 25^th^ and 75^th^ percentiles. The whiskers are the interquartile range (i.e., Q3-Q1) augmented by 50%, and the symbols are outliers.

A series of ANOVAs were also run to assess the effect of Group (i.e., musicians vs. nonmusicians) on the cognitive variables tested. Given the significant difference between the two groups in terms of years of education and vocabulary score (which might affect cognitive measures), these variables were entered in the analyses as covariates. Descriptive statistics and scores’ distributions are presented in Table 2 and Fig 3.

**Table 2:** Descriptive statistics (M and SD) for the cognitive measures of interest by group (musicians vs nonmusicians).

	Musicians		Nonmusicians	
*Measure*	M	SD	M	SD
Corsi Forward	6.10	0.97	5.10	2.17
Corsi Backward	4.55	0.83	6.65	2.39
VPTA	7.50	1.15	5.55	1.88
LST	17.30	1.49	11.05	3.36
Intrusion LST	0.06	0.06	0.18	0.23
sMRT	4.40	1.39	2.20	2.19
sEFT	7.85	1.39	5.90	3.09

Note: VPTA: Visual Pattern Test Active; LST: Listening Span Test; sMRT: short Mental Rotations Test; sEFT: short Embedded Figures Test.

#### Short-term memory tasks

The main effect of Group was not significant for the Forward Corsi task, *F*(1,36) = 2.24, *p* = .14, *η*^*2*^_*p*_ = .06 (the adjusted means for musicians and nonmusicians were, respectively, *M*_adj_ = 6.25 and *M*_adj_ = 4.95, SE = 0.51, after correcting for the covariates), nor were the main effects of the covariates (*Fs* < 1). The main effect of Group was not significant for the Backward Corsi task either, *F*(1,36) = 1.72, *p* = .20, *η*^*2*^_*p*_ = .05 (*M*_adj_ = 5.01 vs *M*_adj_ = 6.19, SE = 0.53, for musicians and nonmusicians respectively, after correcting for the covariates), nor were the main effects of the covariates (*Fs* < 1.48, *p*s > .23).

#### Complex working memory tasks

A significant main effect of Group emerged for the visuospatial WM task, the VPTA, *F*(1,36) = 18.85, *p* = .001, *η*^*2*^_*p*_ = .34), with musicians scoring higher than nonmusicians (*M*_adj_ = 8.16 vs. *M*_adj_ = 4.89, SE = 0.44, respectively, after correcting for the covariates). The main effects of the covariates were not significant (*F*(1,36) = 1.59, *p* = .22, *η*^*2*^_*p*_ = .04, for years of education; and *F*(1,36) = 2.08, *p* = .16 *η*^*2*^_*p*_ = .06 for vocabulary).

A significant main effect of Group was also found for the verbal WM task, the LST, *F*(1,36) = 50.36, *p* = .001, *η*^*2*^_*p*_ = .58, with musicians scoring higher than nonmusicians (*M*_adj_ = 18.34 vs *M*_adj_ = 10.01, *SE* = 0.69, respectively, after correcting for the covariates). As for the covariates, the main effect of years of education was not significant, *F* < 1, but a significant main effect of vocabulary emerged, *F*(1,36) = 8.71, *p* = .006, *η*^*2*^_*p*_ = .20.

A significant main effect of Group was found for intrusion errors (a measure of inhibition) in the LST, *F*(1,36) = 13.12, *p* = .001, *η*^*2*^_*p*_ = .27, with musicians making fewer intrusion errors than nonmusicians (*M*_adj_ = -0.03 vs *M*_adj_ = 0.26, *SE* = 0.05, respectively, after correcting for the covariates). The main effects of the covariates were not significant (*F*(1,36) = 2.62, *p* = .11, *η*^*2*^_*p*_ = .07 for years of education, and *F*(1,36) = 2.36, *p* = .13, *η*^*2*^_*p*_ = .06 for vocabulary).

#### Visuospatial tasks

A significant main effect of Group emerged for the sEFT, *F*(1,36) = 24.18, *p* < .001, *η*^*2*^_*p*_ = .40: musicians had higher scores than nonmusicians (*M*_adj_ = 9.25 vs *M*_adj_ = 4.98, *SE* = 0.57, respectively, after correcting for the covariates). As for the main effects of the covariates, years of education was not significant, *F*(1,36) = 1.97, *p* = .170, *η*^*2*^_*p*_ = .05, while vocabulary was significant, *F*(1,36) = 13.73, *p* = .001 *η*^*2*^_*p*_ = .28.

For the sMRT too, the main effect of Group was significant, *F*(1,36) = 10.22, *p* = .003, *η*^*2*^_*p*_ = .22: musicians had higher scores than nonmusicians (*M*_adj_ = 4.72 vs *M*_adj_ = 1.88, *SE* = 0.52, respectively, after correcting for the covariates). As for the covariates, here again, the main effect of years of education was not significant, *F*<1, but the main effect of vocabulary was significant, *F*(1,36) = 5.86, *p* = .02, *η*^*2*^_*p*_ = .14.

### Composite measures

To examine briefly in a synthetic way how musicians and nonmusicians differed in terms of their auditory and cognitive aspects, we computed two composite measures: one for the auditory and the other for the cognitive tasks. Principal component analyses were run for this purpose, extracting the first component of all auditory variables and the first component of all cognitive variables (i.e., Forward Corsi task, Backward Corsi task, VPTA, LST, intrusion errors in LST, sEFT, and sMRT). The first component of auditory performance had an eigenvalue = 1.77, and accounted for 29% of the variance. The first component of cognitive performance had an eigenvalue = 3.57, and accounted for 51% of the variance. The results of ANCOVA (entering vocabulary and years of education as covariates) confirmed that the two groups differed in cognitive performance, which was better in musicians than in nonmusicians, *F*(1,36) = 34.11, *p* < .001, *η*^*2*^_*p*_ = .49. Among the covariates, vocabulary had a significant (and positive) main effect, *F*(1,36) = 7.81, *p* = .008, *η*^*2*^_*p*_ = .18, while years of education did not, *F*(1,36) = 1.66, *p* = .206, *η*^*2*^_*p*_ = .04. The two groups also differed in auditory performance, which was again superior in musicians, *F*(1,38) = 17.53, *p* < .001, *η*^*2*^_*p*_ = .32 (consistently with the previous analysis, vocabulary and years of education were not entered as covariates in this case).

#### Discriminating power of the variables

We also examined how the variables observed–as well as the composite measures–could help discriminate between musicians and nonmusicians. This analysis was limited to the variables for which significant differences had emerged between the two groups. Taking this approach, variables are treated as a group “discrimination test”. This analysis provides a direct measure of how a given variable can help to discriminate between participants belonging to different groups, without making any assumptions on the variables’ distribution. For each variable, a receiver operating characteristic (ROC) analysis was conducted, and the area under the curve (AUC) was calculated as its performance as a classifier of group. The true-positive rate was calculated with regard to “musicians” (whereas the false positive rate concerned “nonmusicians”).

**Fig 4:**
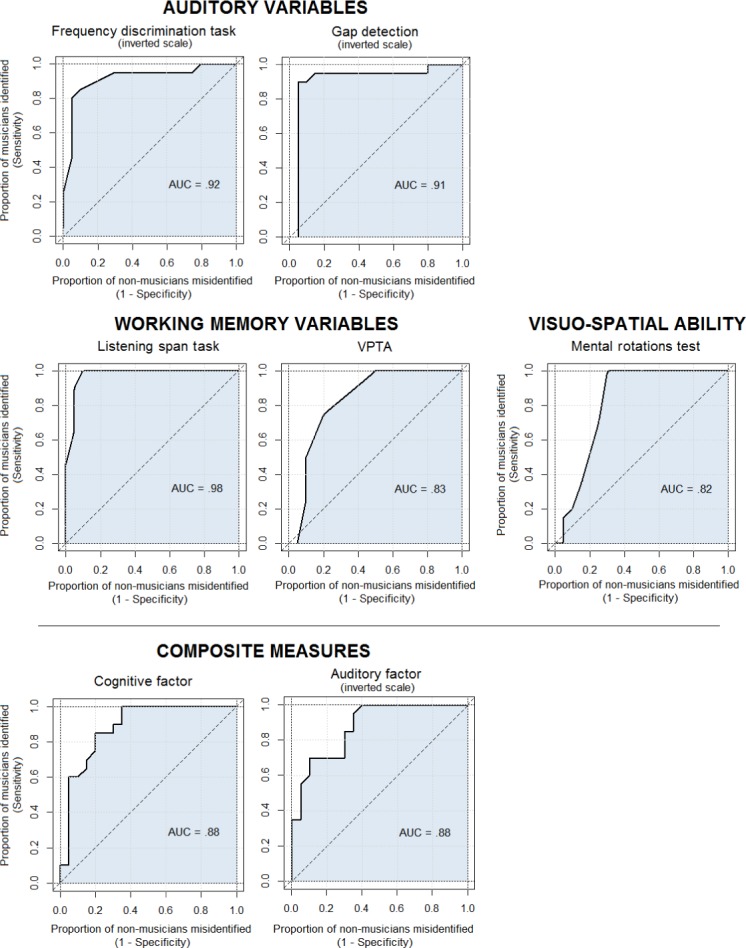
ROC curve (and AUCs) for variables used as group classifiers. The true positive rate refers to the proportion of musicians correctly identified. Variables given on an “inverted scale” indicate that they increasingly differentiate musicians from nonmusicians as their value decreases (i.e., musicians have lower values than nonmusicians). Note that, in the auditory domain, lower scores correspond to a better performance.

Following the accuracy classification proposed by Zhu and colleagues [93], we considered an AUC > .80 as good, and an AUC > .90 as excellent. Fig 4 shows the ROC curves (with the AUCs) for the variables measured with a good classification power (i.e., AUC > .80). The variables reported on an “inverted scale” indicate that musicians could be discriminated from nonmusicians using a lower value as the criterion. Among the auditory variables, frequency discrimination and gap detection emerged as excellent classifiers (with an AUC = .92 and .91, respectively). As for the cognitive, in particular the memory variables, the VPTA emerged as a good classifier for identifying musicians (AUC = .83), and the LST emerged as an excellent classifier (AUC = .98). Always among the cognitive, visuospatial variables, the sMRT emerged as a good classifier (AUC = .82). Finally, as regards the composite measures, both cognitive and auditory performance emerged as good group classifiers (both AUCs = .88).

#### Correlation between the cognitive and auditory domains

Correlations were tested considering the composite measures, but no significant correlations emerged: auditory performance did not correlate with cognitive performance in either musicians (*r* = -.06, *p* = .81) or nonmusicians (*r* = -.23, *p* = .36). Finally, the correlations partialized for vocabulary and years of education did not vary within each group.

## Discussion

The aim of the present study was to investigate the auditory and cognitive profiles of elderly (> 65 years old) but still active professional musicians, with a lifelong music training, comparing them with those of nonmusicians of similar age. To the best of our knowledge, this is the first study to have investigated auditory skills and cognition together in older musicians, using a multivariate design, and numerous indicators for the construct examined.

The results of the auditory tasks revealed a similar performance in musicians and nonmusicians on pure-tone audiometry. This test is often regarded as a measure of the peripheral auditory system’s integrity, and consequently of the efficiency of peripheral auditory processing. Our two groups obtained comparable results, as seen in previous studies (e.g. [40]). None of our participants showed evident signs of hearing loss, but they all had the typical audiometric pattern of older age, characterized by presbycusis [41]. On the other hand, the musicians performed better than the nonmusicians in terms of auditory abilities. This was true of four of the six abilities investigated in the present study. The musicians did better in frequency and duration discrimination tasks, but not in intensity discrimination and profile analysis. The musicians’ advantage in the frequency and duration discrimination domains is well documented in the literature (e.g. [10, 11, 40]). Unfortunately, as far as we are aware, nobody has investigated the intensity discrimination and profile analysis in musicians and nonmusicians. A possible explanation for our failure to find a difference in this dimension may come from the perceptual learning literature. In perceptual learning, participants’ discriminatory abilities are improved by means of dedicated laboratory training sessions, during which they are asked to perform a psychophysical task repeatedly. Such training usually improves participants’ performance in the trained task. It seems from the literature, however, that performance can be improved in frequency and duration discrimination tasks, but not in intensity discrimination tasks [94].

As concerns cognitive abilities, although the musicians in our sample had fewer years of formal education than the nonmusicians and scored worse in a vocabulary test, they performed better in the complex WM and visuospatial tasks, while the two groups did not differ in the two short-term memory measures. In other words, consistently with previous studies, and with our expectations, the musicians’ and nonmusicians’ performance was similar in the less complex (short-term recall) tasks, which demand fewer resources than WM tasks, and have been found less age-sensitive (e.g. [69]). In contrast, musicians showed a clearly enhanced WM performance, irrespective of (verbal or visuospatial) task content. Taken together with the lack of any significant difference between musicians and nonmusicians on short-term memory tasks, this pattern of findings suggests that the difference in the memory profile of older musicians vis-à-vis other older people concerns memory tasks requiring a high level of control, and this aspect would have a role whatever the task content. The fact that task content was not crucial in the case of WM tasks is indirectly in line with the similar age-related decline seen in both verbal and visuospatial WM tasks [60]. Musicians’ better performance in complex WM tasks supports the findings of previous studies, which identified this mechanism as a prime candidate for musicians’ broader underlying cognitive advantages in young adult age [95], with the novelty that our results extend these findings to older musicians too. The larger education of nonmusicians in comparison to nonmusicians needs to be explained. Such a difference can be regarded as normal among Italian older adults. The old Italian conservatory could be started at any age after age 13 (i.e. immediately after completing the first cycle of secondary school). The conservatory lasted ten years. Many conservatory students did not attend the second cycle of secondary school simultaneously with the conservatory. In addition, talented musicians who started playing in childhood were not inclined to take any other types of school after beginning the conservatory. In other words, fewer years of formal education in older musicians are not surprising.

Musicians also revealed a more efficient inhibition than nonmusicians, as emerged from their fewer intrusion errors in the LST. This might be because music playing involves the need to ignore some misinformation such as, for example, when performing in a music ensemble where each musician plays a different melody. The repeated practice in this peculiar condition might enhance the attentional control ability of musicians. This hypothesis is supported by studies showing activation of the frontal executive areas when people listen to music [96–98]. This stronger cognitive control of our older musicians is consistent with other reports [64, 65], and may also account for their enhanced WM performance. Efficient inhibitory mechanisms correlate with efficient WM functioning because the latter is of limited capacity, and the former would only allow relevant information to occupy WM capacity (e.g. [99]).

Musicians also performed better than nonmusicians in higher-order cognitive tasks that involved multi-step image manipulation (in the sEFT) and mental rotations (in the sMRT). Our findings are in line, for the sMRT at least, with [32], and newly extend this knowledge to older adult musicians. The musicians’ greater accuracy in these two visuospatial tasks might be due to their having better visualization and sensorimotor skills than other older adults thanks to years of daily practice with their musical instrument, and/or to their greater experience and proficiency with sight-reading (they need to maintain longer visual sequences in their WM while performing). For example, rapid score-reading while playing a musical instrument is a task that involves visualization and sensorimotor abilities. This type of training may enhance the ability of the musician to manage complex visuospatial patterns and to memorize them. In other words, the training of playing an instrument while reading a score, may improve performance in visuospatial tasks requiring spatial manipulation of stimuli. This supports the notion that musicians’ retention of their musical and sight-reading skills throughout their adult lives would be associated with better performance in higher-order visuospatial tasks. Music training thus seems related to enhanced skills in higher-order cognitive processes, not in the more simple ones.

The between-group differences seen in both the auditory and the cognitive domains were further confirmed when the discriminatory power of the variables was examined in relation to the group. In particular, both the composite measures obtained with our principal component analysis emerged as good group classifiers, with an identical discriminatory power (AUC = .88). Examining the specific variables, frequency discrimination and gap detection for hearing, and WM and mental rotations for cognitive measures, proved good-to-excellent in pinpointing our (elderly) musicians, as shown by the analyses on the ROC curves. As for the cognitive measures, the distributions of the WM and mental rotation variables overlapped very little between the musicians and the nonmusicians. This means that even the musicians with the poorest cognitive profiles (within their own group) performed as well as, or better than the best-performing nonmusicians. This pattern of results confirms the different profile characterizing older musicians, in terms of their auditory and higher-order cognitive abilities.

Finally, concerning the possible relationship between auditory processing and cognitive processing, the present study revealed no significant link, neither in absolute threshold, i.e., peripheral processing (e.g. [76, 77, 100–102]), nor in supra-threshold, i.e., central processing (e.g. [43, 44, 54]). The musicians performed better in the higher-order cognitive tasks than the nonmusicians, irrespective of their performance in the auditory tasks. This lack of a relationship (often reported in the literature) can be interpreted as follows: in many studies, the strength of the relationship between sensation (including hearing) and cognition was boosted by statistical artefacts, particularly when it was calculated on the performance of participants of very different ages, e.g., young adults vs old adults (see [43, 103] for detailed explanations). After controlling for statistical artefacts, the strength of the relationship is small [43]. Assuming that the strength of the relationship is distributed around a small value, the published results may be biased because studies that found a relationship were more likely to be published than those that did not [104, 105].

Altogether, the present findings show that musicians have better performance than nonmusicians in auditory and cognitive tasks (in particular the complex ones). Here, older musicians retained the advantage over nonmusicians found in previous studies [63–65, 106]. In the present study, moreover, musicians performed better than nonmusicians irrespective of the sensory system tapped by the cognitive test (i.e., regardless of whether the test was presented auditorily or visually).

These results raise the following question: why old musicians should have better auditory and cognitive performances than old nonmusicians? The advantage in auditory tasks is easily explained in the light of the literature on experts’ performance: experts (musicians in the case in point) perform better than non-experts (i.e., nonmusicians) with stimuli they are familiar with, i.e., sounds. What remains to be seen is why musicians here performed better than nonmusicians in cognitive tasks. The literature on this issue is quite controversial especially because of the different criteria used to define and include older musicians (e.g., age of the musicians, years of music training) in the various literature studies. We can hypothesize different types of explanations. On the one hand, there may be some uncontrolled variable typical of quasi experiments such as the current that is responsible for the differences in the cognitive tasks (e.g., related to a different motivation, interest in the tasks executed etc.). Another possibility is that individuals with high cognitive abilities are more likely to become musicians, and that is why musicians perform better than nonmusicians in cognitive tasks. Any of these possible explanations would, presumably, give musicians an advantage over nonmusicians in all tasks, but this was not the case.

The alternative explanation is that a better cognitive performance might be a consequence of having trained to become a musician. According to this hypothesis music training, especially for people like the musicians in our sample, who were still active in their profession, and with a lifetime of music training behind them, may act as a protective factor (contributing to the cognitive reserve [107]) against age-related changes in cognition [108]–and also in some auditory skills [57]–better enabling musicians to retain complex cognitive skills in old age than their nonmusical peers. Their cognitive reserve may help to compensate for the age-related decline in cognition because long-term music training would favor neuroplasticity, which modifies synaptic connections or neural growth processes. Learning a skill may help preserve the gray and white matter structures during the normal process of aging, when the brain generally undergoes substance loss (e.g. [39, 109]).

A possible and alternative aspect behind the benefit of music training could be the notion of mismatch. According to this idea [110], training activities are effective when they induce a “supply-demand” mismatch, i.e., they are sufficiently difficult to exceed the available capacity, but not so difficult to induce disengagement from the task. Being active in music may well be seen as a constant source of supply-demand mismatch: musicians need frequently to learn new musical pieces, or to work in different orchestras. In other words, the superiority of musicians over nonmusicians could be due by being continuously challenged by novel requests.

These speculations need to be confirmed by further studies, which should make an effort to examine and support not only the behavioral plasticity, but also the brain plasticity of older musicians. Our results are in line with studies showing that older adults who engage in cognitively stimulating activities–even in later life–have slower rates of cognitive decline, whatever their early education levels [111]. It remains unclear, however, whether the present findings were driven by our participants’ music training, or by their being individuals more likely to engage in activities generally. The quasi-experimental nature of the present study can shed no light on this issue. Future research, for example, should recruit nonmusicians that, as well as musicians, are still actively engaged in cognitively demanding activities (i.e. related to their previous work or to social activities). It may be the case that an active individual in any domain that requires effortful and different cognitive processes would look like a musician. Such a comparison group would disentangle the role of life-long training from the role of the music training. Nonetheless, the few available experimental studies suggest that even a short-term music training (e.g., singing and listening to music) suffices to improve not only mood but also several cognitive abilities in elderly people suffering from dementia [112].

We would like to acknowledge some of the limitations of the present study. First, there is the small sample size–though it is not easy to find professional musicians still active beyond 65 years of age. The design of our study prevents us from establishing any causal relationships, such as whether it was participants’ music training that enhanced their cognition, or whether other variables were responsible for the differences between the musicians and nonmusicians. By the same token, it is also hard to say whether the effects observed in our sample were due to the participants’ musical experience or to a predisposition to succeed in music as well as in cognitive tasks generally. Even if it was established that music training directly enhances cognition, it would remain unclear whether it may represent a true protective mechanism against cognitive decline in advanced age. Under this hypothesis, the difference in cognitive tasks performance between musicians and nonmusicians should be larger in older than in younger adults (previous studies, however, failed to report an age by music training interaction on performance in specific tasks; see e.g. [113]; c.f. also [114] for conceptually similar results in aircraft pilots). From an applicative point of view, this hypothesis would suggest that even music training provided for the first time in advanced age may be have a beneficial effect on cognitive skills. Only longitudinal research (e.g. [115, 116]), and studies on neural compensation mechanisms, however, may be able to address all these questions. Lastly, we were unable to differentiate between musicians with different types of music training, or who played different instruments. The type of musical instrument is known, for example, to modulate a musician’s ability in certain perceptual tasks [117]; and orchestra conductors have greater spatial tuning skills than pianists or nonmusicians [4]. Whether the instrument played also modulates musicians’ cognitive performance remains to be seen.

In conclusion, the present study suggests that music training is associated with enhanced auditory and cognitive abilities at any age, and it might be a driver of experience-dependent plasticity in aging–suggesting the importance of providing music training for older adults.
