# Correction: Auditory and cognitive performance in elderly musicians and nonmusicians

**DOI:** 10.1371/journal.pone.0192918

**Published:** 2018-02-08

**Authors:** Massimo Grassi, Chiara Meneghetti, Enrico Toffalini, Erika Borella

There are errors in the Author Contributions. The correct contributions are: Conceptualization: MG EB. Data curation: MG ET EB. Formal analysis: MG ET EB. Methodology: MG EB. Supervision: MG CM EB. Writing-original draft: MG CM ET EB.

There are errors in the Funding section. The correct funding information is as follows: This study was conducted when Massimo Grassi was supported by the grant CPDA123458 ("Progetto di ricerca di Ateneo"), and Chiara Meneghetti by the grant CPDA140302 ("Progetto di ricerca di Ateneo") awarded by the University of Padova. The Department of General Psychology financially supported Erika Borella for this study.
